# Advances and Challenges in COVID-19 and Pneumonia

**DOI:** 10.3390/v16030331

**Published:** 2024-02-22

**Authors:** Zichen Ji, Javier de Miguel-Díez

**Affiliations:** 1 Pulmonology Service, Gregorio Marañón General University Hospital, Gregorio Marañón Health Research Institute (IiSGM), 28007 Madrid, Spain; jizich72@gmail.com; 2 Faculty of Medicine, Complutense University of Madrid, 28040 Madrid, Spain

In recent years, the pandemic caused by SARS-CoV-2 has posed a significant challenge to the entire medical community [1]. Since December 2019, the scientific community’s focus has been on understanding the epidemiology and pathophysiology of COVID-19 infection and developing treatments and vaccines against this virus. In addition to COVID-19, there have also been peaks of respiratory infection caused by other microorganisms, including influenza [2], respiratory syncytial virus [3], and mycoplasma [4].

One of the most notable achievements has been understanding the complexity of interactions between respiratory viruses, the lungs, and the immune system [5]. Genomic sequencing techniques have been employed to characterize viral genomes, thereby rapidly identifying new strains of SARS-CoV-2 as they emerge, enabling the analysis of their infectivity and virulence. Similar techniques have also been used on other respiratory viruses, such as influenza. Knowledge of genomic evolution has allowed knowledge about its pathophysiology and the development of vaccines and specific treatments [6].

Research into respiratory virus infections extends beyond the respiratory system to systemic manifestations or complications in other organs [7]. A clear example has been the intensive investigation into the thromboembolic disease predisposed by COVID-19 [8].

Despite these advances, significant gaps in knowledge about SARS-CoV-2 persist. One of the major challenges is the early identification of COVID-19 patients who may develop severe pneumonia [9]. To date, despite the existence of various prognostic scales in COVID-19, none are universally accepted and used in routine clinical practice to the extent of the CURB65 (confusion, blood urea nitrogen, respiratory rate, blood pressure, and age of 65 or older) or Pneumonia Severity Index (PSI) scales [10]. This is due to variability in clinical presentation and the rapid progression of the disease, necessitating the identification of biomarkers and more specific models to better predict the risk of developing severe illness.

Furthermore, the optimal management of pneumonia in the context of SARS-CoV-2 infection remains an area of debate and ongoing study. Optimizing the use of mechanical ventilation [11], the use of antiviral and anti-inflammatory medications, including monoclonal antibodies, and the need for potential adjunctive treatments are crucial topics requiring further in-depth study [12].

In future research, a focus on studies to improve the prevention and treatment of respiratory viruses is important [13]. In the case of SARS-CoV-2, currently available vaccines are already updated to provide protection against the Omicron sub-variant [14]. However, viruses are characterized by constant mutations, and with each newly emerging variant, the effectiveness of these vaccines would need to be studied. Additionally, treatment also needs to be continuously updated.

Research on the immune response in the lungs caused by SARS-CoV-2 infection is crucial, as it helps understand how the immune system contributes to the pathogenesis of pneumonia and thus serves as a basis for new, more precise, and effective therapeutic strategies [15].

The application of new technologies, such as big data analysis and artificial intelligence, could revolutionize the epidemiological study and identification of risk factors and patterns of clinical presentation [16]. The use of these tools could improve prognostic accuracy and decision making for better disease management.

In conclusion, the scientific community has made a tremendous effort to address the challenge of the COVID-19 pandemic. Broad knowledge has been generated in just a few years, although questions requiring further investigation to improve the management of respiratory virus diseases remain unresolved.
